# Zanubrutinib-induced aseptic meningitis: a case report and literature review

**DOI:** 10.3389/fphar.2023.1242491

**Published:** 2023-08-31

**Authors:** Jinjun Yang, Lian Wang, Xiao Zhong, Chenlu Yang, Yu Wu

**Affiliations:** ^1^ Department of Hematology and Institute of Hematology, West China Hospital, Sichuan University, Chengdu, China; ^2^ Department of Dermatology, West China Hospital, Sichuan University, Chengdu, China; ^3^ Department of Nuclear Medicine, West China Hospital, Sichuan University, Chengdu, China

**Keywords:** Zanubrutinib, bruton tyrosine kinase, follicular lymphoma, aseptic meningitis, hematological diseases

## Abstract

Zanubrutinib is a Bruton tyrosine kinase (BTK) inhibitor used in B cell malignancy treatment and is generally well tolerated in most patients. Zanubrutinib-induced aseptic meningitis is currently not reported. Herein, we present the first case of zanubrutinib-induced aseptic meningitis. A 33-year-old woman was diagnosed with relapsed/refractory follicular lymphoma and subsequently developed aseptic meningitis after receiving zanubrutinib treatment. We reviewed the literature and uncovered the lack of current reports on zanubrutinib or other BTK inhibitor-induced aseptic meningitis. Moreover, we summarized cases on aseptic meningitis induced by common chemotherapy and targeted drugs used for hematological diseases. Drug-induced aseptic meningitis (DIAM) is a drug-induced meningeal inflammation. The possible pathogenesis is the direct stimulation of the meninges via intrathecal injection of chemotherapy drugs and immune hypersensitivity response caused by immunosuppressive drugs. It is more common in women with immune deficiency and mainly manifests as persistent headache and fever. Cerebrospinal fluid examinations mainly demonstrate a significant increase in cells and proteins. DIAM diagnosis needs to exclude bacterial, fungal, viral, and tuberculosis infections; neoplastic meningitis; and systemic diseases involving the meninges. The prognosis of DIAM is usually favorable, and physicians should detect and stop the causative drug. In conclusion, zanubrutinib-induced aseptic meningitis is a rare but serious complication, and physicians should be promptly aware of this adverse event to avoid serious consequences.

## Introduction

Bruton tyrosine kinase (BTK) is a nonreceptor kinase that plays a crucial role in oncogenic signaling for leukemic cell proliferation and survival in multiple B cell malignancies (Pal Singh et al., 2018). BTK inhibitors form a covalent bond with a cysteine residue (Cys-481) in the kinase domain to inactivate BTK, which further restrains the B-cell antigen receptor pathway activation and blocks malignant B-cell proliferation and survival (Kim, 2019). Zanubrutinib is a next-generation BTK inhibitor that has presented promising antitumor activities in both preclinical models and clinical studies (Syed, 2020). Zanubrutinib monotherapy is generally well tolerated in patients with B cell malignancies. A pooled safety analysis of zanubrutinib reports that the common grade of ≥ 3 nonhematologic treatment-emergent adverse events (AEs) (≥ 2%) are pneumonia, hypertension, upper respiratory tract infection, urinary tract infection, sepsis, diarrhea, and musculoskeletal pain (Tam et al., 2022). A grade of ≥ 3 headache is reported in 1% of patients and with no reports of severe AEs of the central nervous system (CNS). Herein, we report the first case of zanubrutinib-induced aseptic meningitis and performed a literature review of drug-induced aseptic meningitis (DIAM) in hematological diseases. We searched PubMed for articles containing the words “zanubrutinib,” “ibrutinib,” “acalabrutinib,” “tirabrutinib,” “orelabrutinib,” “pirtobrutinib,” “nemtabrutinib,” “Bruton tyrosine kinase inhibitors,” “rituximab,” “cytarabine,” “methotrexate,” “apolizumab,” “dasatinib,” “RG7356,” “daratumumab,” “alemtuzumab,” and “aseptic meningitis” from inception up to May 2023 with no language restrictions.

### Case presentation

**FIGURE 1:**
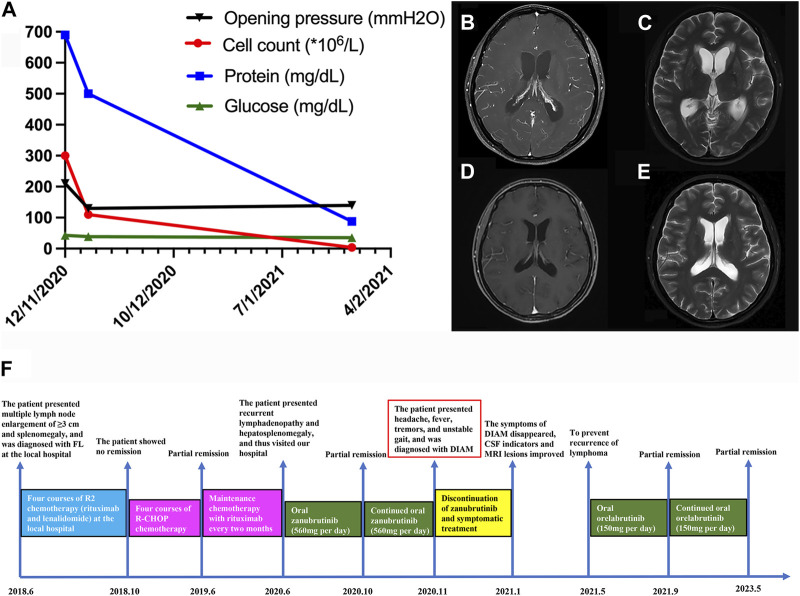
**(A)** Changes in various cerebrospinal fluid indicators before and after zanubrutinib discontinuation in the patient **(B–C)** Brain MRI of the patient before zanubrutinib discontinuation. **(B)** T1 enhancement; **(C)** T2. **(D–E)** Brain MRI after the patient stopped using zanubrutinib for 4 months. **(D)** T1 enhancement; **(E)** T2. **(F)** Detailed time course of the patient’s clinical course and therapeutic regimen.

A 33-year-old Chinese woman presented to our department with the chief complaint of headache, fever, tremors, and unstable gait for 10 days in November 2020. She had a known history of relapsed/refractory follicular lymphoma (FL) for 2 years. Two years ago, she presented with multiple lymph node enlargement and splenomegaly a few months after giving birth. She had no symptoms of fever, night sweats, and weight loss. She visited a local hospital. The left armpit lymph node biopsy indicated FL (grade II). Positron emission tomography/computed tomography (PET/CT) revealed lymph node enlargement of ≥3 cm diameter and increased β-2-[18F]-Fluoro-2-deoxy-D-glucose (FDG) uptake in the neck, armpits, mediastinum, abdominopelvic cavity, and groin. Additionally, the FDG uptake was increased in both breasts, spleen, and multiple bones. Therefore, she was diagnosed with FL (stage IV, grade 2, group A, FLIPI 3 points). Considering the history of giving birth, she received four courses of R2 chemotherapy (rituximab and lenalidomide) for 4 months at the local hospital. However, remission was not achieved; thus, she was prescribed four courses of standard R-CHOP chemotherapy (rituximab, cyclophosphamide, doxorubicin, vincristine, and prednisone) for 8 months, and she achieved partial remission. Afterward, she received maintenance therapy with rituximab every 2 months. However, 5 months ago, she experienced recurrent lymphadenopathy and hepatosplenomegaly, and she visited our hospital. The PET/CT confirmed lymphoma relapse. Considering that new targeted drugs, including obinutuzumab and phosphoinositide 3-kinase inhibitors, have not yet been marketed in China and this patient could not be a candidate in the clinical trial of FL at our hospital because of hepatitis B virus infection, she was scheduled to receive zanubrutinib treatment (560 mg per day) after obtaining informed consent. She re-achieved partial remission after taking zanubrutinib for 4 months, and oral zanubrutinib was continued. However, 10 days ago, the patient developed neurological symptoms, including headache, fever, hand tremors, and unsteady gait. The laboratory examination revealed negative results for serum procalcitonin, C-reactive protein (1,3)-β-D-glucan and galactomannan test, tuberculosis antibody, and interferon-gamma release assay. Then, a lumbar puncture was performed, and the opening pressure of cerebrospinal fluid (CSF) was 210 mmH2O. The cell count, protein, and glucose were 300 × 10^6^/L (normal range: 0–10 × 10^6^/L), 690 mg/dL (normal range: 15–45 mg/dL), and 42.84 mg/dL (normal range: 45–79.2 mg/dL), respectively (Figure 1A). The ink stain, herpesvirus type II DNA, *mycobacterium tuberculosis* DNA, tuberculosis antibody, culture of bacteria, fungi, and *mycobacterium*, exfoliated cells, metagenome, autoimmune encephalitis-related antibodies, paraneoplastic syndrome-related antibodies, and flow cytometry were negative. Brain magnetic resonance imaging (MRI) enhanced scan illustrated meninges thickening, cistern narrowing, third ventricle and bilateral lateral ventricle enlargement and hydrocephalus, and CSF exudation (Figures 1B, C). Zanubrutinib-induced aseptic meningitis diagnosis was highly suspected based on clinical manifestations and laboratory findings. Therefore, zanubrutinib was stopped and mannitol (125 mL, once every 12 h) was given for dehydration. The patient’s neurological symptoms significantly improved, and headache, fever, hand tremors, and unsteady gait disappeared after stopping zanubrutinib for 2 months. The cell count and protein by the CSF analysis significantly decreased (Figure 1A). Enhanced brain MRI scanning revealed that the enhancement degree of intracranial pia mater and hydrocephalus was reduced (Figures 1D, E). We used another BTK inhibitor orelabrutinib to fight the lymphoma and the patient was well tolerated 4 months later. Up to now, the patient’s lymphoma has been well controlled, and no CNS symptoms have reappeared after 3 years of follow-up. The therapeutic course is summarized in Figure 1F.

## Discussion

Herein, we report a Chinese female patient with relapsed/refractory FL who developed aseptic meningitis after receiving zanubrutinib treatment for 4 months. The diagnosis of zanubrutinib-induced aseptic meningitis was established in our case according to the temporal association between zanubrutinib exposure and the development of CNS symptoms, clinical and laboratory findings, and typical MRI of meningitis. Currently, different BTK inhibitors, including ibrutinib, acalabrutinib, zanubrutinib, tirabrutinib, orelabrutinib, pirtobrutinib, and nemtabrutinib, have been approved or used in the later stage of clinical development for B cell malignancy treatment, such as mantle cell lymphoma, chronic lymphocytic leukemia/small lymphocytic lymphoma, Waldenström’s macroglobulinemia, and FL. Among these BTK inhibitors, except for acalabrutinib, which is prone to causing headaches, other BTK inhibitors rarely cause CNS symptoms (Lipsky and Lamanna, 2020). To the best of our knowledge, no cases of zanubrutinib- or other BTK inhibitor-induced aseptic meningitis have been reported, and this is the first case of zanubrutinib-induced aseptic meningitis. We reviewed the literature on the pathogenesis, clinical manifestations, laboratory test and imaging, diagnosis, differential diagnosis, and management of DIAM.

In DIAM, drug intake history is crucial because no specific characteristics are associated with a specific drug. Nonsteroidal anti-inflammatory drugs, intravenous immunoglobulins, antibiotics, monoclonal antibodies, anticonvulsants, vaccines, and intrathecal drugs are common causes of DIAM (Yelehe-Okouma et al., 2018). To date, no study on the mechanism of BTK inhibitor-induced aseptic meningitis has been published. The mechanisms of aseptic meningitis caused by other drugs may include direct meningeal irritation by intrathecal drug injection and immunologic hypersensitivity reaction caused by systemic administration (Yelehe-Okouma et al., 2018). We speculate that the mechanism of zanubrutinib-induced aseptic meningitis may be the immune hypersensitivity response caused by zanubrutinib that inhibits the activation of the B-cell antigen receptor pathway. Moreover, the patient did not develop DIAM with orelabrutinib; thus, we speculated whether it was related to the differences in CNS permeability between different BTK inhibitors. Currently, no study has compared head-to-head CSF concentrations under treatment with different BTK inhibitors. Three studies have reported that CSF concentrations of three BTK inhibitors (ibrutinib, zanubrutinib, and orelabrutinib) were 1.65, 2.94, and 20.10 ng/mL, respectively (Grommes et al., 2017; Song et al., 2021; Zhang et al., 2021). Therefore, high CSF concentrations of both zanubrutinib and orelabrutinib indicate that DIAM may not be related with the differences in CNS penetration between the different BTK inhibitors. In addition, BTK is the only kinase targeted by orelabrutinib (with >90% inhibition) (Dhillon, 2021), which may explain that aseptic meningitis developed with zanubrutinib rather with orelabrutinib. DIAM symptoms mainly include headache, fever, neck stiffness, nausea, photophobia, disoriented, and short-term memory deficits. The CSF analysis showed high cell and protein and normal glucose levels. MRI of the brain appeared to be normal or displayed signs of meningitis. DIAM is an exclusionary diagnosis, which requires exclusions of bacterial, fungal, viral, and tuberculosis infections, neoplastic meningitis, and systemic diseases involving the meninges (Tattevin et al., 2019). DIAM should be distinguished from these diseases, and we summarized the etiology, clinical features, CSF characteristics, treatment, and prognosis of these diseases in Table 1, which helps the doctors detect and identify DIAM promptly in clinical practice. Overall, no significant difference was found in the clinical symptoms between DIAM and other meningitis. The CSF analysis of DIAM showed high cell count, which may be confused with infectious meningitis and neoplastic meningitis. However, the CSF of DIAM does not present any pathogens or tumor cells, which can be distinguished from infectious meningitis and tumor meningitis. DIAM management mainly includes discontinuing the causative drugs and symptomatic treatments. Most patients can recover after stopping medication for approximately 1 week. Physicians must recognize and accurately discontinue relevant pathogenic drugs. Our patient with FL received immunosuppressive therapy, and CSF examination demonstrated a significant increase in cell and protein levels as previously reported. However, her glucose level was slightly lower than normal, and the brain MRI revealed typical signs of meningitis. In addition, her symptoms significantly improved after discontinuing zanubrutinib for approximately 2 months, which was longer than that previously reported.

**TABLE 1:** Main criteria for DIAM differential diagnosis.

		DIAM (Yelehe-Okouma et al., 2018)	Bacterial meningitis (Hasbun, 2022)	Fungal meningitis (Bystritsky and Chow, 2022)	Viral meningitis (Gundamraj and Hasbun, 2023)	Tuberculous meningitis (Mount and Boyle, 2017)	Neoplastic meningitis (Tattevin et al., 2019)	Systemic diseases with meningeal involvement (Tattevin et al., 2019)
Etiology		NSAIDs, antibiotics, IVIG, monoclonal antibodies, and antiepileptic drugs	*Streptococcus* pneumoniae, group B *Streptococcus*, *Neisseria* meningitidis, *Haemophilus* influenzae, and *Listeria* monocytogenes	Cryptococcus neoformans, Cryptococcus gattii, Coccidioides, Histoplasma, Blastomyces, and Sporothrix schenckii	Enteroviruses, herpesviruses, mumps and measles viruses, arboviruses, and lymphocytic choriomeningitis virus	*Mycobacterium tuberculosis*	Leukemia, lymphoma, breast cancer, lung cancer, and melanoma	Sarcoidosis, Behcet’s disease, Sjögren syndrome, systemic lupus erythematosus, and granulomatosis with polyangiitis
Clinical features		Headache, altered consciousness, fever, and myalgia	Fever, neck stiffness, altered mental status, and headache	Headache, fever, cranial nerve, and other focal neurologic deficits	Headache, fever, neck stiffness, and altered mental status	Headache, fever, cranial nerve, and other focal neurologic deficits	Multifocal neurological lesions, cranial nerve palsies, and cognitive impairment	Cranial nerves palsy, meningoencephalitis, cerebral vasculitis, polyneuropathy, convulsions, and pseudotumor cerebri
CSF	Pressure	Elevated	Elevated	Elevated	Normal	Normal or elevated	Elevated	-
	Cells (×10^6^/L)	300	>1,000	Variable	<1,000	100–500	Elevated	>50 or <50
	Cell type	Neutrophils or lymphocytes	Neutrophils	Lymphocytes	Lymphocytes	Lymphocytes	Tumor cells	Neutrophils or lymphocytes
	Protein	Elevated	Elevated	Normal or elevated	Normal or elevated	Elevated	-	-
	Glucose	Normal	Reduced	Normal or reduced	Normal	Reduced	-	-
	Lactates	<3.5 mM	>3.5 mM	-	<3.5 mM	-	-	-
	Other criteria	Sterile	Bacterial culture	Fungal culture, (1,3)-β-D-glucan	PCR, and NGS	*Mycobacterium tuberculosis* culture, acid-fast bacilli staining, tuberculin skin testing, interferon-gamma release assay	-	Primary diseases
Management		Discontinuation of the causative drugs	Ampicillin, ceftriaxone, vancomycin, third-generation cephalosporin, oxacillin, corticosteroids	Amphotericin B, flucytosine, fluconazole, liposomal amphotericin B, itraconazole	Acyclovir	Isoniazid, rifampin, pyrazinamide, streptomycin, ethambutol, corticosteroids	Check-point inhibitors, monoclonal antibodies	Treatment of primary diseases
Time to regression		Within a few days	7–21 days	>4 weeks	7–15 days	>4 weeks	-	-
Outcome		Favorable	Poor	Poor	Favorable	Poor	Poor	-

Abbreviations: DIAM: drug-induced aseptic meningitis; NSAIDs: non-steroidal anti-inflammatory drugs; IVIG: intravenous immunoglobulin; CSF: cerebrospinal fluid; PCR: polymerase chain reaction; NGS: next-generation sequencing.

Despite the lack of studies reporting aseptic meningitis induced by zanubrutinib and other BTK inhibitors except for our case, BTK inhibitors may cause some other serious and rare CNS AEs, including progressive multifocal encephalopathy (PML) and posterior reversible encephalopathy syndrome (PRES) (Zukas and Schiff, 2018). The mechanism may be associated with impaired cellular immunity caused by BTK inhibitors (Fugate and Rabinstein, 2015; Tattevin et al., 2019; Cortese et al., 2021; Zou et al., 2023). To distinguish aseptic meningitis from other rare complications induced by BTK inhibitors, Table 2 summarizes the pathogenesis, clinical manifestations, imaging features, CSF analysis, diagnostic criteria, treatment strategies, and prognosis of these three rare CNS complications to provide information regarding their management. PML is caused by the reactivation of John Cunningham polyoma virus in the presence of cellular immune impairment. PRES involves perfusion imbalance and reversible vascular edema. DIAM is caused by T cell-mediated immune hypersensitivity and meningeal direct inflammation caused by intrathecal injection of antimetabolic drugs. These three complications occur following immunosuppressive drug therapy, and physicians should promptly identify these rare complications and discontinue relevant suspected immunosuppressive drugs.

**TABLE 2:** Clinical characteristics of rare central nervous system complications induced by drugs in hematological malignancies.

Disease	Pathogenesis	Clinical manifestation	MRI of brain	CSF	Diagnostic criteria	Treatment strategy	Prognosis
PML Cortese, Reich, and Nath (2021)	JCV reactivation in the setting of impaired cellular immunity	Cognitive and behavioral abnormalities, sensory and motor deficits, ataxia, aphasia, cortical visual changes, and seizures	Multiple fusion leukoencephalopathies with asymmetric bilateral distribution	JCV DNA positive	Histopathological diagnosis (the triad of multifocal demyelination); JCV DNA of CSF positive, and clinical and imaging features	Antiviral therapies (cytarabine, cidofovir, topotecan, mirtazapine, mefloquine); Immune reconstitution (discontinuation of immunosuppressive therapy, plasma exchange or immunoadsorption, recombinant IL-2, filgrastim, IL-7, PD1 inhibitor, and adoptive immunotherapy with virus-specific T cells)	Unfavorable (high mortality rate, residual neurological disability, recurrent seizures, persistence of JCV, and recurrence)
PRES Geocadin (2023)	Dysregulated perfusion and reversible vasogenic edema	Headache, seizures, encephalopathy, visual disturbances, and focal neurologic deficits	Focal regions of symmetric hyperintensities	Unremarkable	Clinical and imaging features	Discontinuation of immunosuppressive therapies, anticonvulsants, management of hemorrhagic and increased intracranial pressure	Favorable (most patients can recover, with a mortality rate of 3%–6%)
DIAM Yelehe-Okouma et al. (2018)	Immunologic hypersensitivity reaction mediated by T-cell and direct inflammation of the meninges caused by intrathecal injection of antimetabolic drugs	Fever, headache, meningeal signs, altered consciousness, localization signs, seizures, and myalgia	Unremarkable	Frank meningitis with a predominance of neutrophils, normal glucose level, and moderately elevated protein level	The temporal association, clinical manifestations, and laboratory findings, excluding infectious meningitis	Suspected drug discontinuation and symptomatic treatment	Favorable (most patients can recover)

Abbreviations: PML: progressive multifocal encephalopathy; PRES: posterior reversible encephalopathy syndrome; DIAM: drug-induced aseptic meningitis; JCV: john cunningham polyoma virus; MRI: magnetic resonance imaging; CSF: cerebrospinal fluid; IL-2: interleukin 2; IL-7: interleukin 7; PD1: programmed cell death protein 1.

In addition, we reviewed the literature on common chemotherapy and targeted drugs that can induce aseptic meningitis in hematological diseases (Table 3). Currently, except for the case reported herein, 10 cases of DIAM have been reported, including a total of 13 patients (Thordarson and Talstad, 1986; Flasshove et al., 1992; van den Berg et al., 2001; Pease et al., 2001; Lin et al., 2009; Imataki et al., 2014; Vey et al., 2016; Reddy et al., 2018; Kako et al., 2019; Beaumont and Suner, 2020). The median patient age is 33 (interquartile range, 20.3–56.8) years, and the female patients are dominant (7/11, 63.6%, sexes of two patients were not reported). The primary diseases mainly include acute lymphoblastic leukemia (6/13, 46.2%), acute myeloid leukemia (3/13, 23.1%), chronic lymphoblastic leukemia (1/13, 7.7%), multiple myeloma (1/13, 7.7%), severe aplastic anemia (1/13, 7.7%), and FL (1/13, 7.7%). The associated drugs include cytarabine, apolizumab, dasatinib, daratumumab, alemtuzumab, methotrexate, and RG7356. The administration routes include intravenous infusion, intrathecal and subcutaneous injections, and oral administration. Hematologists should be vigilant for the occurrence of DIAM when these drugs are administered.

**TABLE 3:** Summary of all published cases reporting DIAM in hematological diseases.

Reference	Sex/Age	Disease	Drugs	Administration routs	Symptoms	CSF			Brain CT/MRI	Management	Outcome
						Cell count (cells/mm^3^)	Protein (mg/dL)	Glucose (mg/dL)			
Thordarson and Talstad (1986)	F/24	ALL	Cytarabine	IV	Headache, fever, and neck stiffness	126	Normal	Normal	Normal	Discontinuation of cytarabine and empirical antibiotics treatment	Symptoms disappeared on the fifth day
Flasshove et al. (1992)	M/33	ALL	Cytarabine	IV	Symptoms of meningitis	6,600	Increased	-	Normal	Discontinuation of cytarabine and broad specific antibiotics	Symptoms disappeared within 4 days
van den Berg et al. (2001)	F/15	ALL	Cytarabine	IT, IV, and SC	Headache, fever, nausea, and mild nuchal rigidity	1,200	200	19.8	Normal	Discontinuation of cytarabine, empirical antibiotics treatment, and prevention of corticosteroids and clemastin	Symptoms disappeared
Pease et al. (2001)	F/8	ALL	Cytarabine	IV	Headache, fever, photophobia, and neck stiffness	113	55	50	-	Discontinuation of cytarabine and empirical antibiotics treatment	Symptoms disappeared
Lin et al. (2009)	M/65	CLL	Apolizumab	IV	Headache, fever, disoriented, and short-term memory deficits	840 (92% neutrophils)	113	47	Normal	Discontinuation of apolizumab and empirical antibiotics treatment	Symptoms disappeared over a week
Imataki et al. (2014)	F/53	Ph^+^ ALL	Dasatinib	PO	Hypersensitivity on both palms	Increased (predominance of lymphocyte)	-	-	Normal	Discontinuation of dasatinib	Symptoms disappeared
Vey et al. (2016)	-	AML	RG7356	IV	-	-	-	-	-	Discontinuation of RG7356 and steroid administration	Symptoms disappeared over a week
	-	AML	RG7356	IV	-	-	-	-	-	Discontinuation of RG7356 and steroid administration	Symptoms disappeared over a week
Reddy et al. (2018)	F/46	MM	Daratumumab	IV	Headache, numbness in the chin, and tingling in the mouth and lips	46 (79% neutrophils)	24	89	Normal	Discontinuation of daratumumab and Empiric antibiotic therapy	Symptoms disappeared within 4 days
Kako et al. (2019)	M/19	AML	Alemtuzumab	IV	Headache and fever	Increased	-	-	-	Discontinuation of alemtuzumab and empiric antiviral therapy	Symptoms disappeared
	F/27	SAA	Alemtuzumab	IV	Headache and fever	Increased	-	-	-	Discontinuation of alemtuzumab and empiric antiviral therapy	Symptoms disappeared
	M/58	FL	Alemtuzumab	IV	Headache and fever	Increased	-	-	-	Discontinuation of alemtuzumab and empiric antiviral therapy	Symptoms disappeared
Beaumont and Suner (2020)	F/70	Ph^+^ ALL	Cytarabine and methotrexate	IT	Headache	710 (mostly neutrophils)	28	72	-	Discontinuation of IT	Symptoms disappeared within 2 weeks
Current report	F/33	FL	Zanubrutinib	PO	Headache, fever, hand tremors and unsteady gait	300	690	42.84	meninges thickening, cistern narrowing, third ventricle and bilateral lateral ventricle enlargement and hydrocephalus, and CSF exudation	Discontinuation of zanubrutinib	Symptoms disappeared

Abbreviations: DIAM: drug-induced aseptic meningitis; F: female; M: male; ALL: acute lymphoblastic leukemia; CLL: chronic lymphoblastic leukemia; Ph^+^.

ALL: Philadelphia chromosome–positive acute lymphoblastic leukemia; AML: acute myeloid leukemia; MM: multiple myeloma; SAA: severe aplastic anemia; FL: follicular lymphoma; IV: intravenous infusion; IT: intrathecal injection; SC: subcutaneous injection; PO: peros; CSF: cerebrospinal fluid; CT: computed tomography; MRI: magnetic resonance imaging.

This study has some limitations. First, zanubrutinib was not rechallenged to deeply determine this adverse reaction in our patient. Additionally, this is the first case of zanubrutinib-induced aseptic meningitis, and further observation and the mechanism of zanubrutinib-induced aseptic meningitis should be explored in the future.

## Conclusion

Zanubrutinib-induced aseptic meningitis should be considered a potentially serious adverse drug reaction, and whether other BTK inhibitors can cause aseptic meningitis remains unclear. Physicians, especially hematologists, should be aware of this potential AE. Relevant suspicious drugs should be promptly and effectively discontinued when suspected of DIAM because the symptoms of DIAM are severe and most patients can quickly recover after discontinuing the causative drugs.

## Data Availability

The original contributions presented in the study are included in the article/Supplementary Material, further inquiries can be directed to the corresponding authors.
